# Temporal Variations in the Abundance and Composition of Biofilm Communities Colonizing Drinking Water Distribution Pipes

**DOI:** 10.1371/journal.pone.0098542

**Published:** 2014-05-23

**Authors:** John J. Kelly, Nicole Minalt, Alessandro Culotti, Marsha Pryor, Aaron Packman

**Affiliations:** 1 Department of Biology, Loyola University Chicago, Chicago, Illinois, United States of America; 2 Department of Civil and Environmental Engineering, Northwestern University, Evanston, Illinois, United States of America; 3 Pinellas County Utilities Laboratory, Largo, Florida, United States of America; American University in Cairo, Egypt

## Abstract

Pipes that transport drinking water through municipal drinking water distribution systems (DWDS) are challenging habitats for microorganisms. Distribution networks are dark, oligotrophic and contain disinfectants; yet microbes frequently form biofilms attached to interior surfaces of DWDS pipes. Relatively little is known about the species composition and ecology of these biofilms due to challenges associated with sample acquisition from actual DWDS. We report the analysis of biofilms from five pipe samples collected from the same region of a DWDS in Florida, USA, over an 18 month period between February 2011 and August 2012. The bacterial abundance and composition of biofilm communities within the pipes were analyzed by heterotrophic plate counts and tag pyrosequencing of 16S rRNA genes, respectively. Bacterial numbers varied significantly based on sampling date and were positively correlated with water temperature and the concentration of nitrate. However, there was no significant relationship between the concentration of disinfectant in the drinking water (monochloramine) and the abundance of bacteria within the biofilms. Pyrosequencing analysis identified a total of 677 operational taxonomic units (OTUs) (3% distance) within the biofilms but indicated that community diversity was low and varied between sampling dates. Biofilms were dominated by a few taxa, specifically *Methylomonas*, *Acinetobacter, Mycobacterium*, and Xanthomonadaceae, and the dominant taxa within the biofilms varied dramatically between sampling times. The drinking water characteristics most strongly correlated with bacterial community composition were concentrations of nitrate, ammonium, total chlorine and monochloramine, as well as alkalinity and hardness. Biofilms from the sampling date with the highest nitrate concentration were the most abundant and diverse and were dominated by *Acinetobacter*.

## Introduction

The pipes that are used to transport drinking water through municipal drinking water distribution systems (DWDS) are challenging habitats for microorganisms. The transported water generally contains chemical disinfectants such as chlorine or chloramine, as well as very low concentrations of organic carbon and inorganic nutrients [1]. Despite these challenges, microbes frequently colonize the interior surfaces of DWDS pipes [1]. Indeed the pipe surfaces may represent the best possible microbial habitats within DWDS, as previous research has shown that surface attachment can enable bacteria to grow in oligotrophic habitats due to the accumulation of nutrients at the solid-liquid interface [2], [3]. In addition, biofilm formation can provide bacteria with protection against chemical disinfectants [4]–[6].

Relatively little is known about the species composition and ecology of biofilms within DWDS. Obtaining samples from below-ground pipes is difficult and expensive [7], and as a result most of the work that has been done on drinking water biofilms has been based on model systems run in the laboratory [6], [8], [9]. While these studies have provided valuable insight into biofilm formation within drinking water, model systems often differ in significant ways from actual DWDS, including duration of biofilm growth, temporal variability, water flow conditions, diversity of pipe materials and the presence or absence of disinfectants. Additionally, most of the work on microbes within DWDS has focused on classical pathogens such as *Vibrio cholerae* and *Salmonella typhi*, emerging pathogens such as *Campylobacter jejuni* and *Legionella pneumophila*, or indicator organisms for fecal contamination, such as coliform bacteria [1]. Many of these studies have used culture-based techniques [1], which are able to assess only a small fraction of natural microbial diversity [10]. In contrast, recent studies using molecular approaches have demonstrated the predominance of nonpathogenic bacterial species within drinking water biofilms [7], [11], [12].

Information regarding the composition and ecology of biofilms within DWDS is valuable for several reasons. First, it improves our general understanding of microbial life in oligotrophic habitats, including built environments. Secondly, biofilms in DWDS are a concern for public health as they have been shown to harbor and protect pathogens from disinfectants and increase pathogen persistence in DWDS [13]. Thirdly, there is evidence that the activities of nitrifying microorganisms in DWDS can decrease monochloramine concentrations [14], which could lead to increased microbial growth and possibly increased persistence and transport of pathogens [15]. Finally, the presence of biofilms in DWDS can promote pipe corrosion [16] and cause taste and odor problems in the water [3]. Understanding the microbial composition and development of DWDS biofilms can suggest strategies for management of these problems.

We report here the analysis of biofilms found within pipe samples collected five times over an 18 month period from a DWDS in Pinellas County, FL, USA. Sections of below-ground pipe were cut and transported to the lab, and biofilm communities within the pipes were analyzed by heterotrophic plate counts and tag pyrosequencing of 16S rRNA genes.

## Materials and Methods

Pipe samples were collected from the municipal drinking water distribution system in Pinellas County, FL, USA, which is operated by Pinellas County Utilities (PCU). The water supply for this system is a blend of groundwater and treated surface water, with desalinated seawater being used periodically as needed. Before entering the DWDS water is treated at the Tampa Bay Water Treatment Plant (TBW) by a four stage process: 1) clarification using the ACTIFLO system (Kruger Inc., Cary, NC), 2) ozone disinfection, 3) biologically active filtration, and 4) disinfection with chlorine. Disinfection within the PCU DWDS is based on maintenance of a chloramine residual, although the utility does switch to chlorine disinfection for a brief period in the summer each year to limit biofilm growth. The switch to chlorine occurred once during our sampling period, specifically between August 1 and September 11, 2011. The average flow through the system during our sampling period was 54.9 million gallons per month.

Sections of six-inch ductile-iron pipe from the main line in the Seminole, FL region within the Pinellas County DWDS were collected periodically over an 18 month period during planned replacement events. Specifically, pipe sections were collected on February 20, 2011, July 20, 2011, December 20, 2011, March 20, 2012, and August 8, 2012. All pipe samples were collected at approximately 9 am, before peak demand which occurs at approximately 10 am. The water main that we sampled was approximately 40–45 years old. The average water age for the main line during our sampling period was 3 to 4 days and the average velocity was 0.4 c.f.s. The sampling location is downstream of a large above ground storage (AGS) tank that was permanently shut down on June 19, 2012 due to concerns about nitrification occurring within the tank. A key outcome of the shut-down of this water tank was decreased water age at our sampling location for the August 2012 sampling date.

On each sampling date the road surface and soil above the water main were excavated using a backhoe. Soil was cleared from around the pipe by hand using a shovel and any soil adhering to the exterior of the pipe was removed using a brush or cloth. The exterior of the pipe was disinfected by pouring a 10% bleach solution over the pipe and simultaneously wiping the pipe exterior with a cloth saturated with 10% bleach solution. A section approximately 1 ft. long was cut from the 6-inch diameter pipe using a scoring-type pipe cutter. The pipe section was capped at one end using a flexible PVC cap with adjustable clamps. The capped pipe section was filled with dechlorinated water from the same water main, which was collected in a plastic carboy and dechlorinated on-site using sodium thiosulfite (∼2 g/g Cl_2_). The pipe section was filled until overflowing, capped at the other end, placed in a cooler with ice packs, and shipped overnight to Northwestern University, Evanston, IL. Water was also collected from the main for chemical analysis and transported to the Pinellas County Utilities lab in a cooler.

### Sample Processing

In the laboratory one cap was removed from the pipe section and the water was carefully poured out. The interior of the pipe section was rinsed gently with filter-sterilized tap water to remove unattached or settled solids. Biofilm samples were collected from three separate, evenly-spaced sections of the pipe interior. For each section, biofilm material was collected by scraping a 5.5 cm wide band around the entire interior circumference of the pipe with a sterile spatula, resulting in a sampling area of 263.3 cm^2^. The collected biofilm material was transferred to a sterile 10 ml vial and 3 ml ultrapure water was added. The suspension was homogenized by vortexing and large particles such as corrosion byproducts were allowed to settle out of suspension. From this suspension 100 µl was used for the plate count assay (see method below) and 2 ml was used for molecular analysis of the biofilm communities (see methods below). The 2 ml for molecular analysis was transferred to a 2 ml microcentrifuge tube and centrifuged at 10,000×g for 10 minutes. The supernatant was then discarded and the remaining biofilm pellet was stored at -20°C.

### Water Chemistry

All water chemistry analyses were done by Pinellas County Utilities. Analyses were performed based on either EPA Methods or Standard Methods for the Examination of Water and Wastewater [17]. The specific methods used for each assay and results of the water chemistry assays are listed in Table 1. Total haloacetic acid (HAA) concentrations in the source water were determined periodically by EPA method 552.2.

**Table 1:** Water chemistry.

		Sampling Date				
Analyte	Method	February 2011	July 2011	December 2011	March 2012	August 2012
Temperature (°C)	SM 2550 B	20.6	29.1	20.2	23.8	28.5
pH	SM 4500 H-B	7.6	7.58	7.65	7.52	7.69
Total Organic Carbon (mg L^−1^)	SM 5310-C	1.8	1.9	2.2	2	2.3
Total Phosphorous as P (mg L^−1^)	EPA 365.4	0.35	0.24	0.41	0.34	0.31
Nitrate as N (mg L^−1^)	EPA 300.0	0.08	0.11	0.09	0.08	0.43
Nitrite as N (mg L^−1^)^1^	EPA 300.0	BD	BD	BD	BD	BD
Free Ammonia as N (mg L^−1^)	SM 4500 NH3-F	0.19	0.42	0.21	0.16	0.14
Total chlorine (mg L^−1^)	SM 4500 CL-G	2.9	1.6	2.5	3	3
Monochloramine (mg L^−1^)	SM 4500 CL-G	3.2	1.6	2.2	2.6	2.7
Alkalinity as CaCO_3_ (mg L^−1^)	SM 2320 B	170	190	170	180	150
Calcium Hardness (mg L^−1^)	SM 2340 B	210	202	202	205	192
Specific Conductance (umhos cm^−1^)	SM 2510 B	549	521	429	447	477
Aluminum (mg L^−1^)^2^	EPA 200.7-DW	BD	BD	BD	BD	BD
Calcium (mg L^−1^)	EPA 200.7-DW	84.3	80.7	81	81.9	65.1
Iron (mg L^−1^)	EPA 200.7-DW	0.055	0.079	0.381	0.046	0.014
Magnesium (mg L^−1^)	EPA 200.7-DW	7.35	6.51	6.39	6.53	7.14

BD  =  below detection limit.

1 detection limit 0.02 mg L-1.

2 detection limit 0.015 mg L-1.

### Plate Count Assay

R2A agar was purchased as a dried powder (Fisher Scientific, Pittsburgh, PA) and prepared according to the manufacturer's instructions. Biofilm suspensions were serially diluted from 10^2^ to 10^4^ in ultrapure water and 100 µl of all dilutions were spread on R2A agar plates. Plates were incubated at 37°C for 36 hours and counted. Counts were normalized based on the surface area of the pipe from which the biofilm had been collected.

### Molecular Analysis of Biofilm Communities

DNA was extracted from the frozen biofilm pellets using the Power Biofilm DNA Kit (MoBio Laboratories, Carlsbad, CA) according to the manufacturer's instructions and stored at −20°C. For tag pyrosequencing of bacterial 16S rRNA genes the extracted DNA was sent to Research and Testing Laboratory (Lubbock, TX). Polymerase chain reaction (PCR) amplification was performed using primers 530F and 1100R [18]. The 530F primer was chosen in order to obtain sequences for the V4 hypervariable region, which has been shown to provide species richness estimates comparable to those obtained with the nearly full-length 16S rRNA gene [19]. Sequencing reactions utilized a Roche 454 FLX instrument (Roche, Indianapolis, IN) with Titanium reagents. Sequences were processed using MOTHUR software [20]. Briefly, any sequences containing ambiguities or homopolymers longer than 8 bases were removed. Remaining sequences were individually trimmed to retain only high quality sequence reads and sequences were aligned based on comparison to the SILVA-compatible bacterial alignment database available within MOTHUR. Aligned sequences were trimmed to a uniform length of 127 bases and chimeric sequences were removed using UCHIME [21] run within MOTHUR. Sequences were grouped into phylotypes by comparison to the SILVA-compatible bacterial alignment database and algal chloroplast and mitochondrial sequences were removed from the data set. To avoid any biases associated with different numbers of sequences in each of the samples we randomly subsampled a total of 6,808 sequences from each sample, and used these subsampled sequences for all downstream analyses. Sequences were clustered into operational taxonomic units (OTUs) based on 97% sequence identity using the average neighbor algorithm. Rarefaction curves were produced using MOTHUR. The total OTU richness in each sample was calculated based on the Chao1 richness estimator [22]. The diversity of each sample was assessed based on the Shannon index [23] calculated using the Primer software package (Primer V.5, Primer-E Ltd., Plymouth, UK). The community composition of the individual samples was compared by using MOTHUR to calculate distances between sites based on the theta index [24]. The significance of differences in theta index scores between sites was assessed by analysis of molecular variance (AMOVA) run within MOTHUR. PC-ORD v. 6.08 (MjM Software, Gleneden Beach, Oregon, USA) was used to ordinate the theta index distance matrix via non-metric multidimensional scaling (nMDS) and to determine correlations between the water chemistry data and the axes in the nMDS ordination.

### Statistical Analyses

Plate count data and diversity scores were analyzed by one-way analysis of variance (ANOVA) based on sampling date and pairwise comparisons were made by Tukey's post hoc test. Correlations were assessed by determining Pearson product-moment correlation coefficients and Bonferroni-corrected probabilities. Correlation between the relative abundance of *Methylomonas* sequences and the concentration of total HAA in the source water was based on average abundance data for each biofilm sampling date and the HAA concentration from the closest source water sampling date. All statistical analyses were run using Systat 13 (Systat Software, Inc., San Jose, CA) and p values less than 0.05 were considered to be significant.

### Data Sharing

All of the sequence data analyzed in this paper can be downloaded from the National Center for Biotechnology Information (NCBI) Sequence Read Archive (SRA) with accession number SRP038002.

## Results

### Water Source

**Figure 1:**
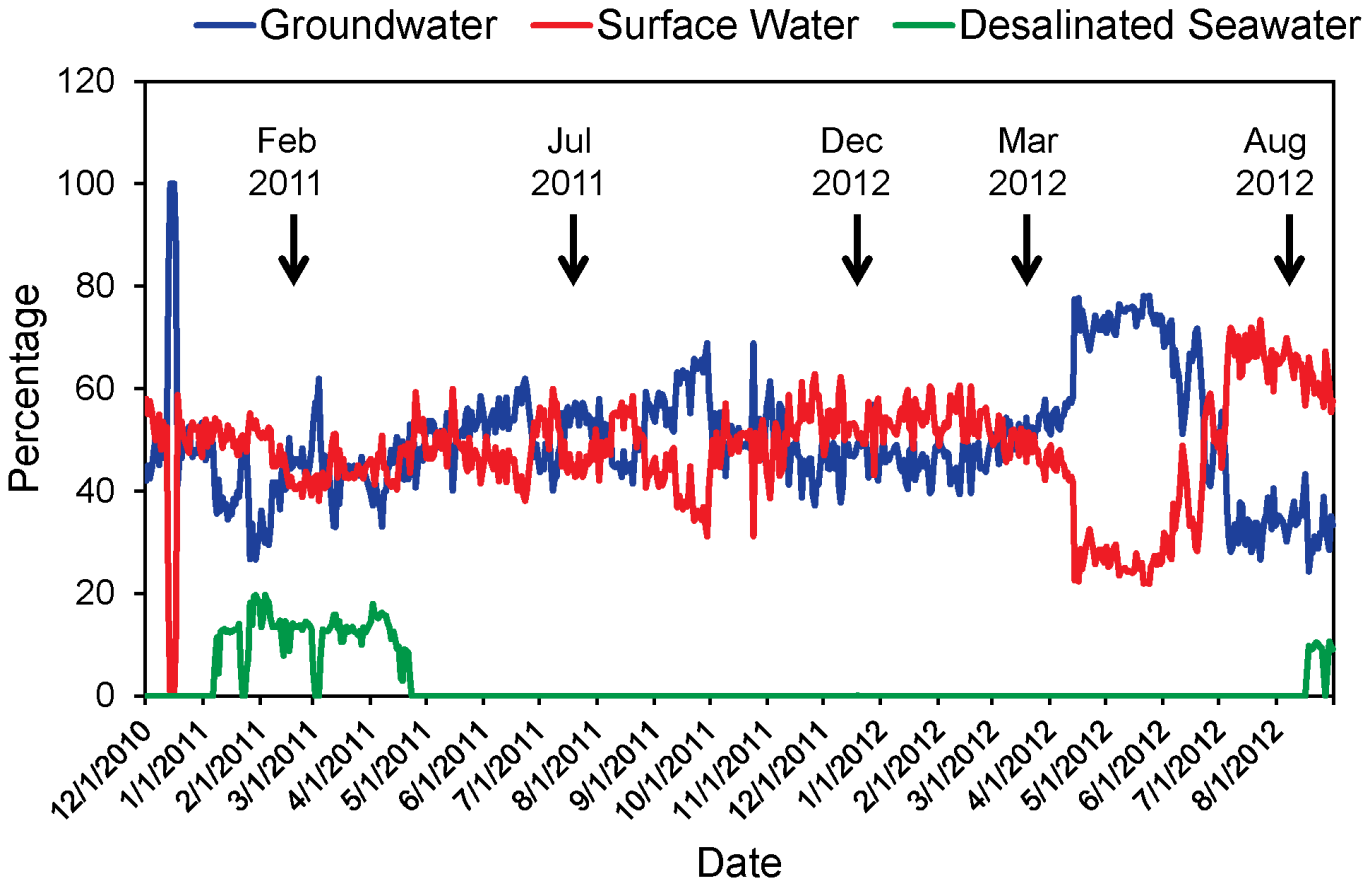
Relative percentages of source waters within PCU drinking water distribution system. Biofilm sampling dates are indicated by black arrows.

The source water for the PCU DWDS is a blend of groundwater, surface water, and desalinated seawater. An approximately equal mix of groundwater and surface water was the most common during the study period, although there were some significant variations in the relative proportions of source waters (Fig. 1). For the four weeks preceding our February 2011 sampling date, the source water averaged 38% Groundwater, 48% surface water and 14% seawater. Between June 20, 2011 and March 20, 2012, a period which included three of our sampling dates, the source water averaged 50% Groundwater, 50% surface water and 0% seawater. Prior to our August 2012 sampling date there were some dramatic shifts in source waters. Between April 14, 2012 and June 9, 2012 the water was predominantly groundwater, with an average of 73% of the water coming from groundwater over that period. Finally, between July 6, 2012 and August 8, 2012 the water was predominantly surface water, with surface water representing an average of 67% of the source water over that period.

### Water Chemistry

**Figure 2:**
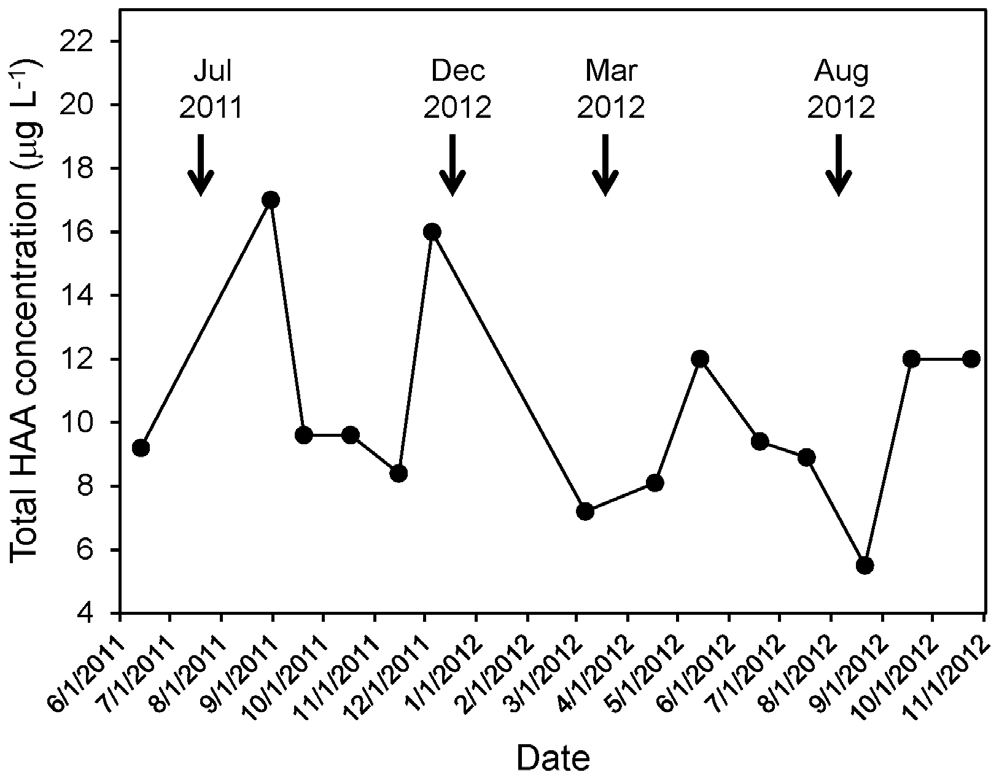
Concentrations of total haloacteic acids (HAA) in the source water for the PCU drinking water distribution system. Biofilm sampling dates are indicated by black arrows.

Water temperature varied seasonally in the water main from which the biofilm samples were obtained, being higher in summer months (July and August) than winter months (February, March and December) (Table 1). Some water chemistry parameters varied with sampling date but did not show seasonal trends. For example, there were large fluctuations in concentrations of nitrate (from 0.08 to 0.43 mg L^−1^), free ammonia (from 0.14 to 0.42 mg L^−1^) and iron (from 0.014 to 0.381 mg L^−1^). The August 2012 sampling date had the highest concentration of nitrate, approximately four to five times higher than all other sampling dates, and the lowest concentration of free ammonia (Table 1). Analysis of the source water from TBW confirmed a high concentration of nitrate in the source water immediately prior to the August 2012 sampling date (data not shown). Analysis of the source water from TBW also showed fluctuations in concentrations of total haloacetic acids over the course of the study (Fig 2).

### Plate Count Assay

Sampling date significantly affected the numbers of bacteria within the pipe biofilms as measured by heterotrophic plate count assay, with bacterial counts varying over 3 orders of magnitude between sampling dates (p<0.001; Table 2). Samples from the summer months (July and August) had significantly higher counts than the winter months (February, March and December), and there was a significant positive correlation between plate counts and water temperature (R^2^ = 0.605; p<0.001). The August 2012 sampling date, which had the highest nitrate concentration, had bacterial counts that were significantly higher than all other sampling dates (p<0.001; Table 2), and there was a significant positive correlation between plate counts and nitrate concentration (R^2^ = 0.799; p<0.001). There was no significant correlation between plate counts and phosphorous concentration (R^2^ = 0.249; p = 0.058). There were also no significant correlations between plate counts and total chlorine (R^2^ = 0.005, p = 0.802) or between plate counts and monochloramine (R^2^ = 0.285, p = 0.548).

**Table 2:** Numbers of heterotrophic bacteria in pipe biofilms based on plate count assay.

Sampling Date	Number of Bacteria (cfu cm^−2^)^1^	
February 2011	215	a
July 2011	10,887	b
December 2011	2,013	a
March 2012	36	a
August 2012	23,167	c

1 Data points represent mean values (n = 3) and data points followed by different letters are significantly different based on ANOVA and Tukey's HSD posthoc test (p<0.05).

### Bacterial Community Analysis

**Figure 3:**
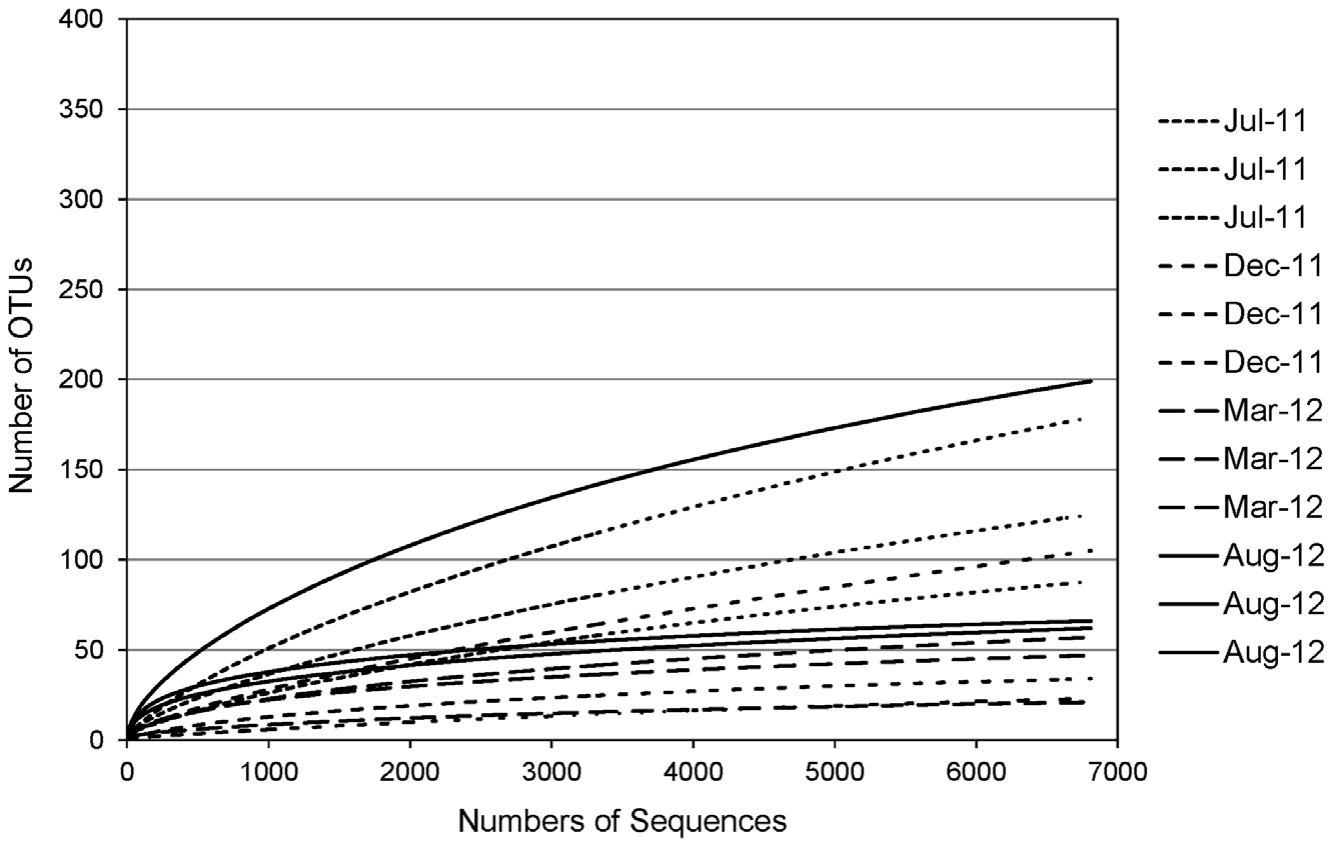
Rarefaction curves for biofilm bacterial communities based on tag pyrosequencing of 16S rRNA genes. OTUs were defined based on 3% distance.

Tag pyrosequencing of 16S rRNA genes was used to profile the bacteria within the biofilms lining the drinking water pipes. Twelve samples representing three replicate biofilm samples from each of four sampling dates (July 2011, December 2011, March 2012 and August 2012) were sequenced successfully. Despite repeated attempts, DNA from the February 2011 pipe samples could not be amplified with the 530F and 1100R primers, so no sequence data were obtained for this sampling date. After processing, the data set included a total of 159,604 high-quality sequence reads. There was significant variation in the number of sequences obtained for each of the samples, from a low of 8,785 to a high of 35,854. To avoid biases associated with unequal numbers of sequences, 6,808 sequences were randomly selected from each of the twelve samples using the subsample command in MOTHUR, producing a total of 81,696 high quality sequences that were used for all analyses of community composition. With this subsampled data set, rarefaction curves for all samples had reached plateaus (Fig. 3), suggesting that the sequencing depth obtained in this study was adequate to capture most of the diversity within these communities. Similarly, a comparison of the total number of OTUs observed in each sample and the estimated total number of OTUs present in each sample demonstrated that for all samples more than 50% of the estimated total number of OTUs in each sample were detected (Table 3).

**Table 3:** Comparison of the number of observed and estimated bacterial OTUs in pipe biofilm communities based on 16S tag pyrosequencing data.

Sampling Date	Observed OTUs^1^	Estimated OTUs^2^	Percent Coverage^3^
July 2011	88	158	55.7%
July 2011	179	332	53.9%
July 2011	125	239	52.3%
December 2011	34	42	81.0%
December 2011	105	194	54.1%
December 2011	23	38	60.5%
March 2012	47	67	70.1%
March 2012	57	87	65.5%
March 2012	21	26	80.8%
August 2012	199	297	67.0%
August 2012	66	74	89.2%
August 2012	62	75	82.7%

1 OTUs were defined based on 3% distance.

2 Total OTUs per sample were estimated based on Chao1 richness estimator.

3 Percent coverage was calculated by dividing the number of observed OTUs by the number of estimated OTUs.

**Figure 4:**
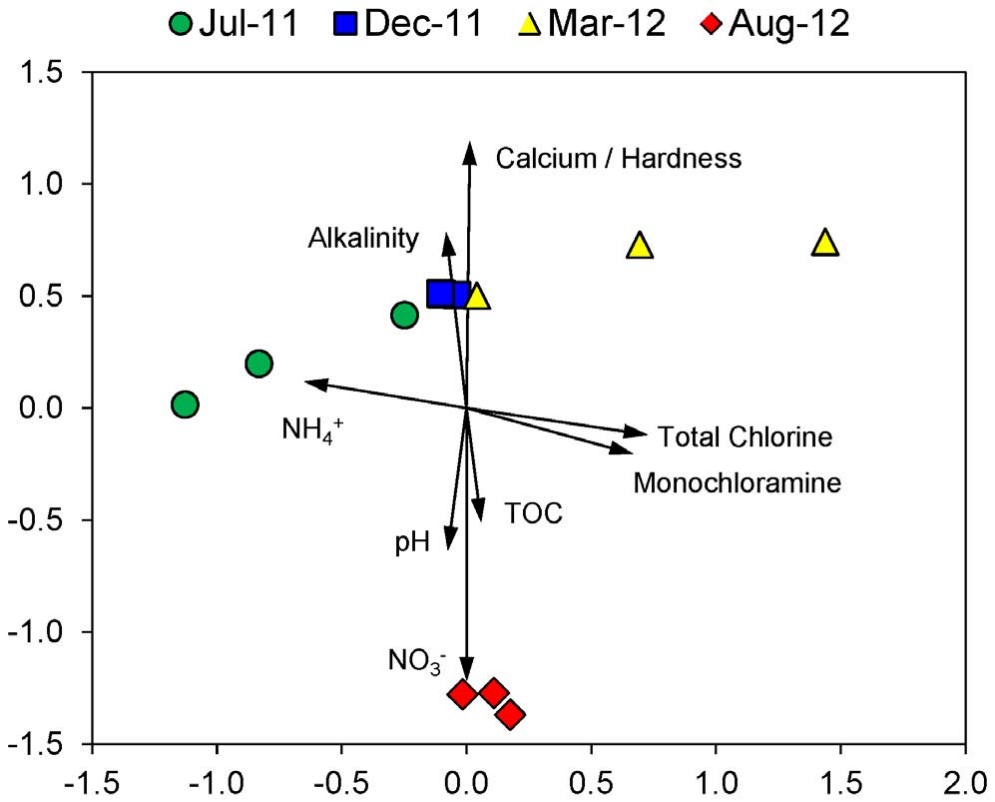
Non-metric multidimensional scaling ordination of biofilm bacterial communities based on tag pyrosequencing of 16S rRNA genes. Stress value of ordination is 0.129. Vector lines represent correlations between physical and chemical variables and the ordination axes. Variables with correlation values less than 0.5 for both axes are not shown.

nMDS ordination (Fig. 4) and AMOVA analysis (Table 4) indicated that there were significant differences between the biofilm bacterial communities from the different sampling dates. Biofilm bacterial communities from August 2012 were the most distinct and were significantly different from the communities from all other sampling dates (Fig. 4 and Table 4). The nMDS ordination also revealed relationships between the community composition and water chemistry parameters (Fig 4). The composition of the biofilm bacterial community from August 2012 was positively correlated with nitrate concentration and negatively correlated with calcium concentration and hardness. The August 2012 communities were also positively correlated with pH and total organic carbon concentration and negatively correlated with alkalinity. In addition, the separation of the biofilm communities from July 2011 and March 2012 on the nMDS ordination was correlated with the concentrations of free ammonia, total chlorine and monochloramine (Fig. 4). Finally, the nMDS ordination demonstrated that there was variation in bacterial community composition between replicates from both the July and March sampling dates, whereas the December and August samples showed a high degree of similarity between replicates (Fig. 4).

**Table 4:** AMOVA analysis of 16S tag pyrosequencing data from pipe samples.

Comparison	p value
August-July	<0.001
August-December	<0.001
August-March	0.0497
July-December	<0.001
July-March	0.0500
December-March	0.0487

### Bacterial Community Diversity

**Figure 5:**
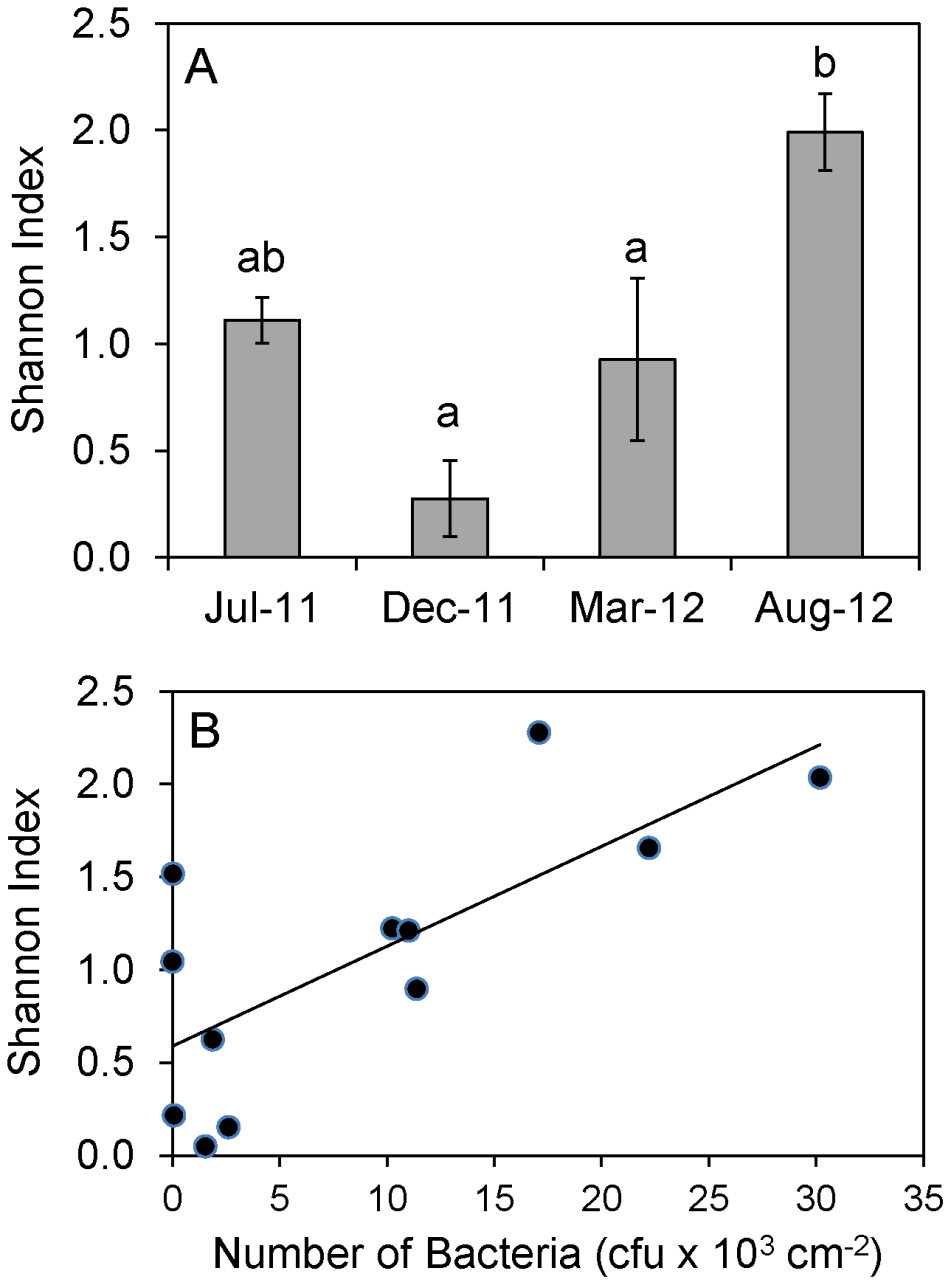
Diversity of bacterial biofilm communities and relationship between bacterial abundance and diversity. (A) Shannon index scores calculated using 16S rRNA gene tag pyrosequencing data. Each data point represents mean value (n = 3) with standard error bars. ANOVA indicated a significant effect of sampling date (p<0.01). Data points with different letters are significantly different based on Tukey's posthoc test (p<0.05). (B) Relationship between biofilm bacterial abundance and diversity. Linear regression indicated R2 = 0.541. Pearson correlation analysis indicated a significant correlation between numbers of bacteria and diversity concentration and resistance (p<0.01).

Pyrosequencing analysis identified a total of 677 OTUs (3% distance) within these biofilms, and the number of OTUs per sample ranged from 21 to 199 (Table 3). Despite this large number of OTUs, the diversity of these communities was low, with three of the four sampling dates showing Shannon index scores below 1.2 (Fig 5A). The Shannon diversity index scores for the biofilm bacterial communities varied significantly between sampling dates (p<0.01) with the biofilms from August 2012 being the most diverse (Fig. 5A). There was also a significant positive correlation (p<0.01) between bacterial abundance in the biofilms and the diversity of the bacterial communities (Fig 5B). As indicated by the low diversity index scores, all of the biofilm communities were dominated by a small number of OTUs, with the ten most abundant OTUs accounting for 93% of the total sequences in the data set.

### Bacterial Community Taxonomic Composition

Analysis of pyrosequencing data indicated that sequences corresponding to the genus *Methylomonas* were the most abundant within the biofilm communities, accounting for 41% of the sequences in the total data set (Table 5). Other abundant sequences corresponded to the genera *Acinetobacter*, *Mycobacterium*, *Pseudomonas*, and *Methylobacterium*, as well as an unclassified genus from the family Xanthomonadaceae and an unclassified genus from the class Betaproteobacteria. The relative abundance data indicated some commonalities between the biofilm bacterial communities from the four sampling dates (Table 5). For example, *Methylomonas* accounted for more than 30% of the sequences for three of the four sampling dates. However, the data also illustrate that there were dramatic differences in the composition of biofilm communities from the different sampling dates, as *Methylomonas* accounted for more than 95% of the sequences in December 2011, but less than 2% in August 2012. A previous study suggested a link between *Methylomonas* bacteria in drinking water and HAA [25], and there was a significant positive correlation between the relative abundance of *Methylomonas* sequences in our biofilms and the concentration of total HAA in the source water (R^2^ = 0.962; p = 0.027). *Acinetobacter* abundance also varied significantly between sampling dates, accounting for 74% of the sequences in August 2012 biofilms, but representing less than 0.1% of the sequences from the other sampling dates.

**Table 5:** Relative abundance of most numerically dominant bacterial genera^1^.

Bacterial genus	All Samples	July 2011	December 2011	March 2012	August 2012
*Methylomonas*	40.8	33.6±15.6	95.5±3.5	32.4±21.3	1.3±0.6
*Acinetobacter*	18.5	0.0±0.0	0.0±0.0	0.0±0.0	74.2±2.4
*Mycobacterium*	14.7	59.1±14.3	0.2±0.2	0.1±0.1	0.0±0.0
Unclass. Xanthomonadaceae	13.5	0.0±0.0	2.6±2.4	50.4±24.4	0.1±0.1
*Pseudomonas*	4.7	0.6±0.3	0.3±0.2	15.5±8.6	2.1±0.2
Unclass. Betaproteobacteria	2.3	0.3±0.1	0.1±0.1	0.0±0.0	9.0±1.6
*Methylobacterium*	1.2	3.7±2.3	0.2±0.2	0.1±0.0	0.8±0.1
Unclass. Bacteria	1.0	0.5±0.2	0.3±0.1	0.0±0.0	3.2±2.8
*Massilia*	0.9	0.0±0.0	0.0±0.0	0.0±0.0	3.4±0.7
Unclass. Gammaproteobacteria	0.4	0.7±0.4	0.2±0.2	0.7±0.5	0.0±0.0

1 Values for each sampling period represent mean values (n = 3) ± standard error.

Sequences corresponding to several genera containing pathogenic species were detected in the biofilms. In a total of 81,696 sequences from all four sampling dates, there were 18 *Escherichia* sequences, 5 *Clostridium* sequences and 3 *Streptococcus* sequences detected, so these genera were extremely rare. *Mycobacterium* represented 59% of the sequences in July 2011, while representing less than 1% of sequences from the other sampling dates. The genus *Mycobacterium* includes two well-known human pathogens, *M. tuberculosis* and *M. leprae*, although these species are generally not found in the environment [26]. The genus *Mycobacterium* also includes a large number of non-pathogenic or occasionally pathogenic species [26]. *Acinetobacter* accounted for 74% of the sequences in August 2012 but less than 1% of sequences from the other sampling dates. Bacteria from the genus *Acinetobacter* are a common cause of nosocomial infections among immunocompromised patients, with the most common example being respiratory infections of ventilated patients [27]. Due to the short length of the sequences obtained in our study, we were unable to discriminate any of the sequences down to the species level, so it is unclear whether the sequences from any of these genera represented pathogenic or non-pathogenic organisms.

The pipes analyzed in this study were ductile iron and did not show significant corrosion or the presence of tubercles. The pyrosequencing data identified only a handful of sequences corresponding to genera known to contain iron-oxidizing species: *Acidovorax* (1 sequence), *Aquabacterium* (1 sequence) and *Thiobacillus* (4 sequences) [28], [29]. In addition, no sequences corresponding to any known ammonia oxidizing bacterial genera were detected. However, a few sequences (19 total) from a known nitrite oxidizing genus, *Nitrospira*, were detected, and all of these sequences were found in the July 2011 samples, which also showed the highest concentration of free ammonia (Table 1).

## Discussion

Pipe samples were collected from the same region of a drinking water distribution system in Pinellas County, FL on five dates over an 18-month period between February 2011 and August 2012. Water from these pipes showed seasonal variations in temperature and some large fluctuations in concentrations of nitrate, free ammonia and iron. The August 2012 sample had a much higher concentration of nitrate and a lower concentration of free ammonia relative to other sampling dates. The shut-down of the upstream above ground storage tank prior to the August 2012 sampling date probably contributed to the observed differences in water chemistry between the sampling dates. The above ground storage tank was shut down because of nitrification occurring in the tank, which could have contributed to the high nitrate and low free ammonia concentrations in the August 2012 sample. The August 2012 samples also had a different source-water mixture than other dates: for one month prior to our sampling date the source water was composed of a higher percentage of surface water and a lower percentage of groundwater than typical for this system. Analysis of the TBW source water at its point of entry to the DWDS confirmed the high nitrate and low alkalinity of the source water at the time of our August 2012 sampling, indicating that source water was a key driver of the unique aspects of the August 2012 water chemistry.

The abundance of bacteria in the biofilms varied greatly across the sampling dates, with higher bacterial numbers in the summer months and lower bacterial numbers in the winter months. These differences in abundance may have been driven by the strong seasonal differences in temperature in the system, as the water in the summer months was on average 7.3°C warmer than in the winter months, and a temperature change of this magnitude can significantly increase the growth rates of mesophilic bacteria [30]. The connection between bacterial abundance in the biofilms and water temperature was further supported by a statistically significant correlation between these two parameters. The differences in bacterial abundance may also have been related to the availability of inorganic nutrients in the drinking water, specifically nitrogen. We found a significant correlation between bacterial cell numbers and nitrate concentrations. Other studies have indicated that inorganic nutrients can be a limiting factor for bacterial growth in DWDS [1]. In our samples, nitrate concentrations were higher in the summer months than in the winter months and were highest in the August 2012 sample. Finally, our results indicated no significant relationship between the abundance of bacteria within the biofilms and the concentrations of total chlorine or monochloramine. These results suggest that low concentrations of chlorine disinfectants may not be effective at limiting biofilm growth within DWDS, owing to the protection provided by the biofilm matrix, as has been demonstrated by previous studies [4], [5], [6]. It should be noted that we assessed bacterial abundance within the pipe biofilms using heterotrophic plate counts. While the limitations of plate counts as estimates of bacterial abundance are well known [31], this method is commonly used to assess bacterial loads in DWDS, so we chose to use this method to make our results comparable to existing data.

Tag pyrosequencing analysis revealed a total of 677 OTUs in the biofilm bacterial communities, with the estimated total numbers of bacterial OTUs per sample ranging from 26 to 332. A previous pyrosequencing survey of drinking water meters revealed a similarly high number of total bacterial OTUs [7]. Despite the large numbers of OTUs observed in our pipe biofilms, these communities were not very diverse, with all samples having Shannon index scores below 2. These low diversity scores reflect the fact that these communities were dominated by a small number of taxa, specifically *Methylomonas*, *Acinetobacter* and *Mycobacterium*. The diversity of the bacterial biofilm communities was significantly correlated with bacterial abundance, suggesting that when conditions within the DWDS were favorable for bacterial growth (e.g. higher temperature and higher nitrate concentrations) a wider range of bacterial taxa were able to proliferate within the biofilms, whereas when conditions were not favorable (e.g. lower temperature and lower nitrate concentrations), a more limited range of bacteria were able to persist. Within our data set the August 2012 samples seemed to represent the most favorable conditions for biofilm growth, as this sampling date had the highest nitrate concentration, one of the highest water temperatures, and supported biofilms with the highest bacterial numbers and the highest bacterial diversity.

*Methylomonas* was the most numerically dominant bacterial genus within the biofilms, with its sequences accounting for more than 40% of all of the sequences recovered. *Methylomonas* is a genus of type I methanotrophic bacteria, which obtain their carbon and energy from the oxidation of methane. *Methylomonas* sequences have been detected previously in drinking water [32], and a recent study of water meter biofilms also detected sequences corresponding to the family *Methylococcaceae*, the bacterial family that includes the genus *Methylomonas*, [7]. *Methylobacterium*, a genus of methylotrophic bacteria that oxidize methyl compounds such as methanol but cannot metabolize methane, was also one of the most commonly detected taxa in our biofilms, although it represented just over 1% of the total sequences. Methylotrophic bacteria, specifically *Methylophilus*, were also detected in biofilms within drinking water meters [7]. The factors favoring high abundance of methanotrophic and methylotrophic taxa within drinking water distribution systems are unclear. We would not expect high concentrations of methane or methanol in drinking water, however these compounds could be produced in anoxic sites within DWDS via anaerobic processes such as methanogenesis or fermentation. Another process that might have supported the growth of methylotrophic bacteria is utilization of haloacetic acid, which is a common by-product of chlorination of drinking water. A recent study isolated a *Methylobacterium* strain from a DWDS biofilm that was capable of growth with haloacetic acid as the sole carbon source [25]. The results of our study, which showed a significant correlation between the relative abundance of *Methylomonas* sequences and HAA concentration in the source water, lends further support to this hypothesis.

Our results demonstrated that there was significant variation in the taxonomic composition of the biofilm bacterial communities within the pipe sections across our sampling dates. nMDS and AMOVA analyses indicated that the August 2012 bacterial communities were the most distinct in terms of their composition, and further examination of the composition of these biofilms revealed that the August 2012 communities were dominated by *Acinetobacter* sequences (74% of total sequences), while *Acinetobacter* sequences represented less than 0.1% of sequences from the July, December and March samples. *Acinetobacter* is a genus of Gram-negative, heterotrophic bacteria [33] that is commonly found in soils and groundwater [34]–[36]. *Acinetobacter* is also one of the most common groups of bacteria isolated from drinking water [1], and a number of *Acinetobacter* species have been shown to produce biofilms [37]–[39]. Therefore the presence of *Acinetobacter* in the pipe biofilms was not surprising. However, the dramatic variation in *Acinetobacter* abundance that we observed between August 2012 and the other sampling dates was remarkable. There were several unique features of the August 2012 sampling date that may have contributed to its distinct biofilm composition. First, the source of the drinking water within the distribution system changed prior to August 2012 to predominantly groundwater for a period of three weeks, with this period of groundwater dominance occurring approximately two months prior to the August 2012 sampling. Since *Acinetobacter* are regularly detected in soil and groundwater, this switch to a groundwater dominated system prior to August 2012 might have provided an additional inoculum of *Acinetobacter* that were able to become established within the pipe biofilms. Another related feature of the August 2012 samples was that the drinking water at that sampling time had a much higher nitrate concentration (at least four times higher) than all of the other sampling dates. The nMDS analysis indicated that nitrate concentration was one of the main drivers of the composition of the bacterial communities within the August 2012 biofilms. The August 2012 biofilms also showed bacterial counts that were more than two times higher than any of the other sampling dates, suggesting more biofilm mass which could have generated more anoxic microsites. Although bacteria from the genus *Acinetobacter* are generally aerobes, there are some species within the genus that can utilize nitrate as an electron acceptor when oxygen is not present [40]. In contrast, *Methylomonas*, which was one of the predominant taxa in July 2011, December 2011 and March 2012 but was less than 2% of total sequences in August 2012, is strictly aerobic [41]. Therefore, the higher drinking water nitrate concentration and possibly more anoxic microsites caused by higher bacterial biofilm growth on the August 2012 sampling date may have provided *Acinetobacter* with a competitive advantage over *Methylomonas*.

*Mycobacterium* abundance also varied considerably over time, as this genus represented 59% of the sequences from the July 2011 sampling date but less than 1% for all other sampling dates. *Mycobacteria* are frequently detected in DWDS and are considered a significant public health issue [42]. The genus *Mycobacterium* consists of approximately 100 species, including a large number of species that are either non-pathogenic or pathogenic under certain situations [26]. For example, nontuberculosis *Mycobacterium* are a major cause of opportunistic infections in immunocompromised hosts [42]. Mycobacteria have been detected previously in this DWDS via culturing, and *M. gordonae* and *M. intracellulare* were the most frequently detected *Mycobacterium* species [43]. *M. gordonae* is among the most frequently reported mycobacteria in drinking water and in DWDS [26] and is generally considered non-pathogenic [44]. *M. intracellulare*, which is part of the *Mycobacterium avium* complex (MAC), has also been detected in DWDS [26]. MAC is the group of non-tuberculosis *Mycobacterium* most commonly associated with human disease, causing primarily pulmonary infections in individuals who are immunocompromised [45]. In this study, we were unable to discriminate the *Mycobacterium* sequences down to the species level, so it is unclear whether the sequences we detected represented potentially pathogenic species.

Several characteristics of *Mycobacteria* enhance their survival in DWDS, including their ability to grow under oligotrophic conditions [46], form biofilms and resist chlorine disinfection [26]. Several recent studies have detected species related to *Mycobacterium* in chlorinated drinking water [47]–[49]. Previous work at the DWDS considered here indicated that the frequency of detection of *Mycobacteria* increased when the disinfectant was switched from chlorine to chloramine in 2002 [43], suggesting that *Mycobacteria* might be less sensitive to chloramine than chlorine. However, a recent study using a model DWDS observed that the relative sensitivity of *Mycobacterium avium* biofilms to chlorine and monochloramine depended on the pipe material [50]. Specifically, *M. avium* was more sensitive to chlorine than chloramine when biofilms were grown on copper pipe, but the reverse was true for *M. avium* biofilms on iron [50], possibly due to corrosion products interfering with free chlorine [51]. Here we found that *Mycobacterium* sequences were most abundant in biofilms from the July 2011 sampling date, which also had the highest level of free ammonia and the lowest levels of total chlorine and monochloramine. These constituents are related, as monochloramine is reductively dehalogenated to ammonia, and monochloramine is the major component of total chlorine in this system. The results of our nMDS analysis indicate that the monochloramine concentration strongly influenced bacterial community composition for the July 2011 sampling date. Therefore, the fact that *Mycobacterium* was predominant only on the sampling date with the lowest level of monochloramine suggests that within the ductile iron pipes in this DWDS monochloramine significantly reduced the abundance of *Mycobacterium* within the biofilms.

An unclassified genus from the family Xanthomonadaceae also varied considerably in abundance across the sampling dates, representing 50% of the sequences from the March 2012 samples but only a small fraction of the sequences from the other sampling dates. The Xanthomonadaceae are obligate aerobic chemoorganotrophs, and this family includes some well-known plant pathogens [33]. Organisms from the family Xanthomonadaceae family were not detected in a previous pyrosequencing survey of drinking water biofilms [7], but these organisms have been isolated from drinking water and from drinking water pipe biofilms using culture based techniques [52]. In this study, we were unable to discriminate the Xanthomonadaceae sequences down to the genus or species level, and the reason for their high abundance in the March 2012 samples is unclear.

The biofilm communities from July 2011 and March 2012 showed much higher variation in composition among replicates than did biofilm communities from December 2011 and August 2012. The December and August samples were each dominated by a single bacterial genus (*Methylomonas* in December and *Acinetobacter* in August) with very little variation between replicates. These data suggest that the environmental conditions in December and August each favored one specific bacterial genus that dominated all of the biofilms on that sampling date. In contrast, biofilms from July and March had several dominant bacterial genera that showed high variations between the replicates, suggesting that conditions during those months produced greater variability by enabling several genera to compete for dominance within the biofilms.

Nitrification is a significant concern in drinking water distribution systems that use chloramine as the secondary residual, as nitrification can lead to a decrease in the chloramine residual, an increase in the growth of heterotrophic bacteria and an increase in concentrations of nitrate and nitrite, which pose risks to human health [53]. Multiple studies have identified nitrifying bacteria in DWDS [53]–[55]. No sequences corresponding to any known ammonia oxidizing bacterial genera were detected in the biofilms analyzed in this study. However, a few sequences from a known nitrite oxidizing genus, *Nitrospira*, were detected in the July 2011 samples, but not in samples from any of the other sampling dates. July 2011 also showed the highest concentration of free ammonia (at least two times higher than all other sampling dates) and unpublished data from PCU confirm that there was a peak in nitrification activity in June and July of 2011. The nMDS analysis indicated that ammonia concentration was a significant driver of the composition of the biofilm bacterial communities from July 2011, and previous studies have indicated that the presence of free ammonia is the principal cause of nitrification in DWDS [56]. Therefore, these data suggest that the high free ammonia concentration combined with the high temperature in July 2011 enabled nitrification to occur within the pipes; however, the lack of detection of ammonia oxidizing bacteria suggests that ammonia oxidation within the biofilms may have been driven by ammonia oxidizing archaea (AOA). Previous studies have detected AOA in drinking water distribution systems [54], but archaea would not have been detected by the bacterial primers used in the current study.

In summary, the results of our study demonstrate that the biofilms within the DWDS pipes were dominated by a few bacterial taxa, specifically *Methylomonas*, *Acientobacter* and *Mycobacterium*, and that the dominant taxa within the biofilms varied dramatically between sampling times. It is likely that these differences in dominant taxa were driven by differences in environmental conditions, and our analysis suggests that nitrate, ammonium, total chlorine, and monochloramine concentrations were key drivers of biofilm bacterial community composition.

Another possibility is that these differences in dominant taxa could have been the result of the founder effect, which stipulates that the founding member of a biofilm will have an advantage over subsequent colonizers and will remain dominant. The founder effect has been suggested as a possible driver of biofilm community composition in a variety of habitats [57]–[60] and could have been a contributing factor to the differences in dominant community members in our biofilms. Further experimental work, which is ongoing in our lab, will be needed to explore the relative contributions of environmental factors and founder effects on the composition of biofilms within drinking water distribution systems.
